# Early molecular events during retinoic acid induced differentiation of neuromesodermal progenitors

**DOI:** 10.1242/bio.020891

**Published:** 2016-10-28

**Authors:** Thomas J. Cunningham, Alexandre Colas, Gregg Duester

**Affiliations:** Development, Aging, and Regeneration Program, Sanford Burnham Prebys Medical Discovery Institute, La Jolla, CA 92037, USA

**Keywords:** Embryonic stem cells, Neuromesodermal progenitors, *Raldh2* knockout embryos, Retinoic acid target genes, *Nkx1-2*, *Zfp503*, *Zfp703*, *Gbx2*, *Id1*, Retinoic acid response elements

## Abstract

Bipotent neuromesodermal progenitors (NMPs) residing in the caudal epiblast drive coordinated body axis extension by generating both posterior neuroectoderm and presomitic mesoderm. Retinoic acid (RA) is required for body axis extension, however the early molecular response to RA signaling is poorly defined, as is its relationship to NMP biology. As endogenous RA is first seen near the time when NMPs appear, we used WNT/FGF agonists to differentiate embryonic stem cells to NMPs which were then treated with a short 2-h pulse of 25 nM RA or 1 µM RA followed by RNA-seq transcriptome analysis. Differential expression analysis of this dataset indicated that treatment with 25 nM RA, but not 1 µM RA, provided physiologically relevant findings. The 25 nM RA dataset yielded a cohort of previously known caudal RA target genes including *Fgf8* (repressed) and *Sox2* (activated), plus novel early RA signaling targets with nearby conserved RA response elements. Importantly, validation of top-ranked genes *in vivo* using RA-deficient *Raldh2^−/−^* embryos identified novel examples of RA activation (*Nkx1-2*, *Zfp503*, *Zfp703*, *Gbx2*, *Fgf15*, *Nt5e*) or RA repression (*Id1*) of genes expressed in the NMP niche or progeny. These findings provide evidence for early instructive and permissive roles of RA in controlling differentiation of NMPs to neural and mesodermal lineages.

## Abstract (precis)

**Summary:** The findings here demonstrate that the signaling molecule retinoic acid (RA) plays an early role in determining how embryonic progenitor cells decide to differentiate into either mesodermal or neural tissues.

## INTRODUCTION

Retinoic acid (RA) is a potent and widely used signaling cue that stimulates differentiation of stem/progenitor cells *in vitro*. The function of endogenous RA in control of stem/progenitor cell differentiation *in vivo* is much less understood (Cunningham and Duester, 2015). This knowledge is essential to provide valuable basic insight into how RA normally controls development and how it may be most useful in regenerative medicine applications.

During development of vertebrate embryos, separation of the three germ layers (embryonic ectoderm, mesoderm and endoderm) is nearly complete by late gastrulation when the previously multipotent epiblast stem cells have differentiated into mostly lineage-restricted progenitors, including progenitors in the caudal epiblast that will give rise to the trunk and tail regions of the embryo. However, a subset of progenitors in the caudal lateral epiblast (and later in the tailbud), known as neuromesodermal progenitors (NMPs), remain bipotent as they can differentiate into either posterior neuroectoderm or presomitic mesoderm in a coordinated fashion to generate the spinal cord and somites for an extended period of time during body axis extension (Tzouanacou et al., 2009; Kimelman, 2011; Henrique et al., 2015; Tsakiridis and Wilson, 2015). NMPs are thus distinct from the neural progenitors that form anterior neural tissue (i.e. forebrain/midbrain). NMPs are unique in that they co-express *Sox2* and *T (Brachyury)* that drive the neuroectodermal lineage and the mesodermal lineage, respectively (Martin and Kimelman, 2012; Olivera-Martinez et al., 2012; Tsakiridis et al., 2014; Wymeersch et al., 2016), along with *Nkx1-2* which is a marker of all caudal progenitors (Delfino-Machin et al., 2005; Tamashiro et al., 2012; Sasai et al., 2014). A positive-feedback FGF and WNT signaling loop maintains an undifferentiated state in caudal progenitors that promote body axis extension (Ciruna and Rossant, 2001; Aulehla et al., 2003; Dunty et al., 2008; Naiche et al., 2011; Martin and Kimelman, 2012; Olivera-Martinez et al., 2012; Jurberg et al., 2014; Cunningham et al., 2015b). Recent studies have shown that NMPs require Wnt signaling for maintenance and differentiation to the presomitic mesodermal lineage (Wymeersch et al., 2016). *In vitro* derivation of NMPs from embryonic stem cells (ESCs) treated with FGF and WNT agonists have recently enabled study of NMPs in precise cellular and molecular detail (Gouti et al., 2014; Turner et al., 2014; Lippmann et al., 2015). The signals and genes that control NMP maintenance and differentiation are currently being investigated.

RA activity is first detected in vertebrate embryos during late gastrulation [embryonic day (E) 7.5 in mouse], just prior to commencement of body axis extension, extending from the posterior hindbrain to the caudal epiblast where NMPs reside (Sirbu et al., 2005; Uehara et al., 2009). Thus, ESCs (which are derived at E3.5) are not normally exposed to RA. The initial source of RA is presomitic mesodermal cells that express *Rdh10* (retinol dehydrogenase 10) and *Raldh2* (*Aldh1a2*, retinaldehyde dehydrogenase) which together metabolize retinol to RA (Duester, 2008; Niederreither and Dollé, 2008). RA functions as a diffusible ligand for nuclear RA receptors (RARs) that bind RA response elements (RAREs) as heterodimers with the nuclear receptor RXR, allowing direct regulation of transcription (Cunningham and Duester, 2015). Loss of RA synthesis in *Raldh2*^−/−^ embryos results in defects in posterior neurogenesis and somitogenesis, followed by premature termination of body axis extension (Duester, 2008; Niederreither and Dollé, 2008). RA is required for body axis extension during the early phase from E7.5-E9.0 when the caudal epiblast exists, but RA is unnecessary for body axis extension during the late phase (E9.5-E13.5) when the caudal region has been transformed into a tailbud (Cunningham et al., 2011). Loss of RA activity in both mouse and avian embryos results in ectopic anterior expansion of caudal FGF signaling, providing evidence that one function of RA signaling during body axis extension (particularly posterior neurogenesis and somitogenesis) is repression of caudal *Fgf8* (Diez del Corral et al., 2003; Vermot et al., 2005; Sirbu and Duester, 2006; Cunningham et al., 2015a). RA also restricts caudal expansion of *Wnt8a*, providing evidence that RA regulates the FGF-WNT feedback loop that maintains caudal progenitors (Cunningham et al., 2015b). Both *Fgf8* and *Wnt8a* possess nearby conserved RAREs, and recent studies demonstrated that the *Fgf8* RARE functions *in vivo* to repress transcription (Kumar and Duester, 2014; Kumar et al., 2016). Another recent study in mouse embryos showed that RA activity in the neural plate is sufficient to repress caudal *Fgf8* and control normal somite size (Cunningham et al., 2015a), but an instructive role for RA within the NMP niche has not yet been described. Loss of RA activity also leads to an imbalance of cell fates within the NMP niche (increased *Tbx6*+ mesoderm near the primitive streak coupled with reduced *Sox2* expression in the caudal epiblast), strongly suggesting RA influences NMP differentiation (Cunningham et al., 2015a).

No systematic, genome-wide study has been conducted to elucidate endogenous targets of RA signaling during NMP differentiation in a strictly cell-specific and physiological context. Previous studies on RA-treated cell lines have typically searched for RA target genes either very broadly (assessing gene expression changes after long time periods of RA exposure leading to expression changes in secondary targets), very crudely [using supraphysiological 1-10 µM RA concentrations that could yield non-specific effects compared to the average endogenous tissue concentration of ∼25 nM observed *in vivo* (Horton and Maden, 1995; Mic et al., 2003)], or without consideration of cell types that are normally exposed to endogenous RA activity (by exposing ESCs or cancer cell lines to RA). ChIP-seq studies using RAR antibodies have been conducted in ESC-derived embryoid bodies (Moutier et al., 2012) to identify potential RAREs near target genes, yielding >13,000 RAR-RXR binding sites in the mouse genome. Although these results do not reveal the key RA target genes in a given endogenous cell type, they do provide a vital resource when assessing the validity of putative RA targets that arise in succeeding studies.

Here, we define the first responding targets of RA signaling in NMPs using whole genome and functional methodologies *in vitro* and *in vivo*. Our studies provide new mechanistic insight on RA action during body axis extension, and provide evidence that RA signaling is essential for proper differentiation of NMPs to both the neural and mesodermal lineages.

## RESULTS

### Derivation of mouse ESC-derived NMPs and effect of RA treatment

Recently established protocols now enable *in vitro* differentiation of *SOX2*+/*T*+ NMPs from mouse and human ESCs with high efficiency, using defined media that provides WNT and FGF signals at specific time points to recapitulate events in the embryo (Gouti et al., 2014; Turner et al., 2014; Lippmann et al., 2015). This enables posterior neuroectoderm differentiation, in contrast to another recent protocol utilizing dual-SMAD inhibition that yields primarily anterior neuroectoderm (Chambers et al., 2009).

**Fig. 1.**
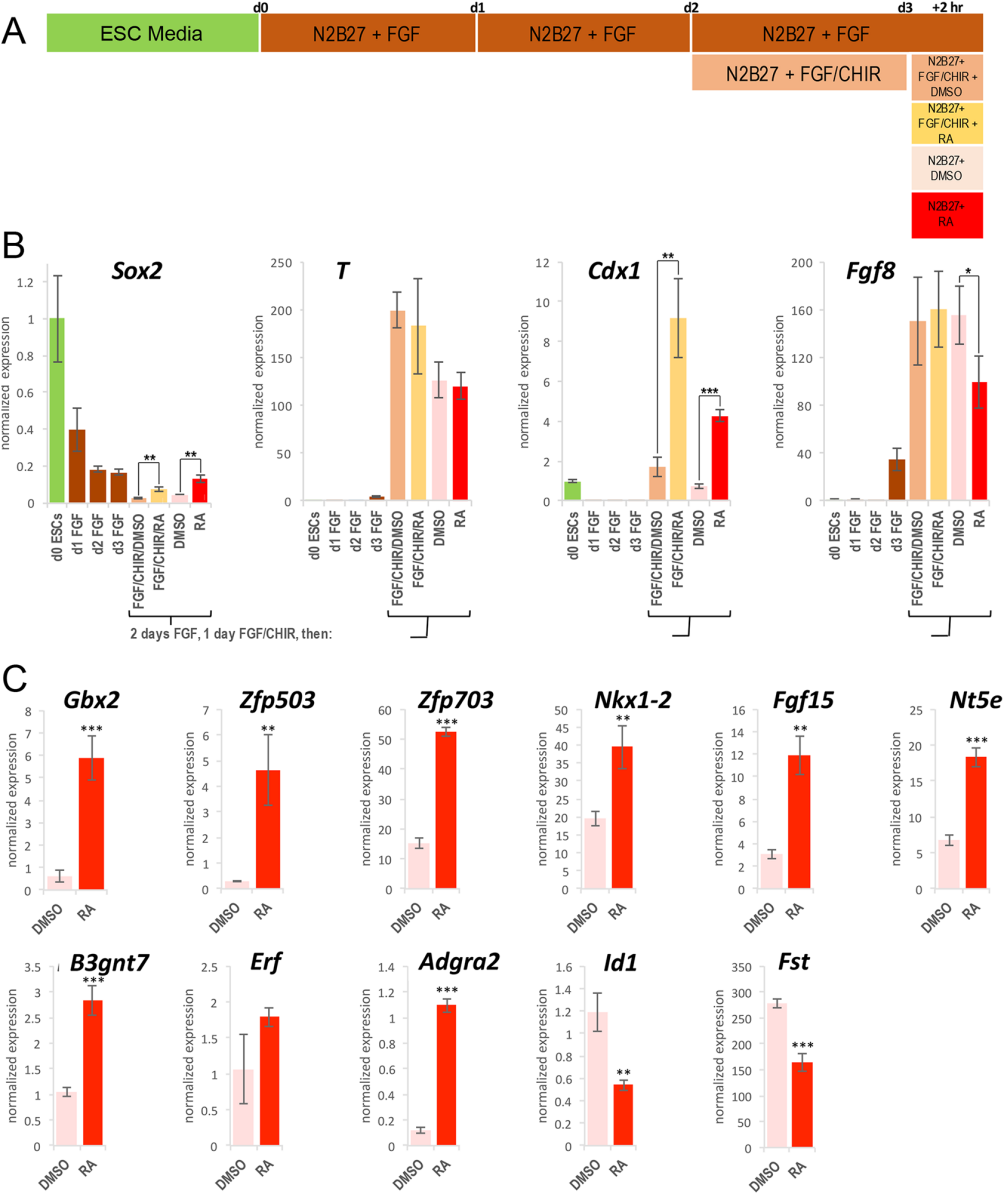
**Derivation of NMPs from mouse ESCs and effect of RA treatment.** (A) The schematic shows the protocol for generating NMPs from ESCs (day 0 to day 3) followed by a further 2 h period in which gene expression is compared with and without 25 nM RA under various conditions. (B) Shown is a 3-day time course of qRT-PCR results used to monitor expression of the key genes co-expressed in NMPs (*Sox2* and *T*) as well as two other genes expressed in caudal progenitors (*Fgf8* and *Cdx1*). (C) The validation of top hits from RNA-seq analysis performed by qRT-PCR analysis of NMPs treated with 25 nM RA or DMSO control for 2 h in the absence of FGF and WNT agonists. Data are expressed as mean±s.e.m. from three replicates (*n*=3); *P<0.05; **P<0.01; ***P<0.001 (Student's *t*-test, two-tailed).

We derived NMPs from mouse ESCs using a protocol similar to that described previously (Gouti et al., 2014; Turner et al., 2014) in which cells were treated for 2 days with N2B27/bFGF, followed by 1 day with N2B27/bFGF/CHIR99021 (CHIR99021 is a WNT agonist herein referred to as CHIR), followed by an additional 2 h treatment under various conditions (Fig. 1A). We adapted the NMP derivation protocol by using N2B27 media with a retinoid-free B27 supplement lacking both RA and any retinoid precursors in order to prevent the cells themselves from generating RA; these conditions mimic the lack of endogenous RA activity in embryos prior to late gastrulation (E7.5) when *Raldh2* expression initiates and RA activity is first observed (Sirbu et al., 2005). In order to assess differentiation to an NMP fate, we examined expression of key NMP markers by qRT-PCR with and without the addition of CHIR that functions as an agonist for WNT signaling, required for activation of *T* to attain the NMP fate (Wymeersch et al., 2016). We found that addition of CHIR resulted in downregulation of the key NMP marker *Sox2* (known to be expressed at a high level in ESCs and a lower level in NMPs) and upregulation of the other key NMP marker *T* that is not expressed in ESCs but is known to be upregulated by WNT in NMPs (Fig. 1B). We also observed that expression of *Fgf8*, known to be activated by WNT caudally (Olivera-Martinez and Storey, 2007; Cunningham et al., 2015b), and *Cdx1*, also known to be activated by WNT caudally (Pilon et al., 2007), were both greatly increased by addition of CHIR. These observations are consistent with the phenotype expected if ESCs were differentiated to a caudal-like NMP fate, and with the phenotype observed in the originally published protocols (Gouti et al., 2014; Turner et al., 2014).

Following the 3 day NMP differentiation protocol, we performed an additional 2 h treatment under five different conditions: bFGF, bFGF/CHIR/DMSO, bFGF/CHIR/RA-25 nM, DMSO, or RA-25 nM as shown in the schematic (Fig. 1A). The 25 nM RA dose represents a physiologically relevant dose for vertebrate embryonic tissues (Horton and Maden, 1995; Mic et al., 2003). We performed qRT-PCR to analyze expression of NMP markers *Sox2* and *T*, as well as *Cdx1* which is known to be directly activated by RA in caudal progenitors (Houle et al., 2003), and *Fgf8* which is known to be directly repressed by RA in caudal progenitors (Kumar and Duester, 2014). *Cdx1* expression was significantly increased by RA (with or without FGF/CHIR) and *Fgf8* expression was significantly decreased by RA in the absence of FGF/CHIR, verifying that our treatment with 25 nM RA for 2 h is sufficient to initiate RA signaling in NMPs for either gene activation or gene repression; inclusion of FGF/CHIR with RA abrogated *Fgf8* repression likely due to CHIR continuing to activate *Fgf8*. Expression of *T* was not significantly changed by RA, whereas *Sox2* expression was significantly increased by RA with or without FGF/CHIR. Our *Sox2* result is consistent with the observation that loss of RA synthesis in *Raldh2*^−/−^ embryos results in reduced *Sox2* expression in caudal progenitors and neural plate (Ribes et al., 2009; Cunningham et al., 2015a), and it provides evidence that upregulation of *Sox2* is part of the early response to RA signaling in NMPs.

### RNA-seq whole transcriptome analysis of RA-treated NMPs

In order to assess the genome-wide effect of RA treatment on mouse ESC-derived NMPs, bFGF/CHIR was removed from the culture medium on day 3 (as shown in Fig. 1A) and NMPs were subjected to the following conditions in N2B27 alone: (i) 2 h 25 nM RA, (ii) 2 h 1 µM RA, or (iii) 2 h DMSO control, with each condition performed in duplicate. Total RNA was collected, purified, and used to conduct whole transcriptome RNA-seq to quantify differentially expressed transcripts. In the absence of RA treatment (DMSO control) several NMP markers were expressed as expected according to average quantitative values for transcript abundance (reads per kilobase of genome mapped per million; RPKM), including relatively low *Sox2* (5.2), high *T* (375.2), moderate *Nkx1-2* (39.11) and *Mlx1* (36.0), while *Sox1* (marking committed neural cells) was very low (0.2). In the presence of RA we observed significantly increased expression of *Rarb* (RPKM increased from 0.1 to 5.0) and *Cdx1* (RPKM increased from 2.7 to 7.0) which are known RA-activated genes in the caudal region of mouse embryos, as well as significantly decreased expression of *Fgf8* isoform f (RPKM decreased from 7.0 to 4.4) which is a known RA-repressed gene in caudal progenitors. These initial observations indicate that RA-treated NMPs are behaving as expected.

### Comparison of NMPs treated with 25 nM RA or 1 µM RA

**Fig. 2.**
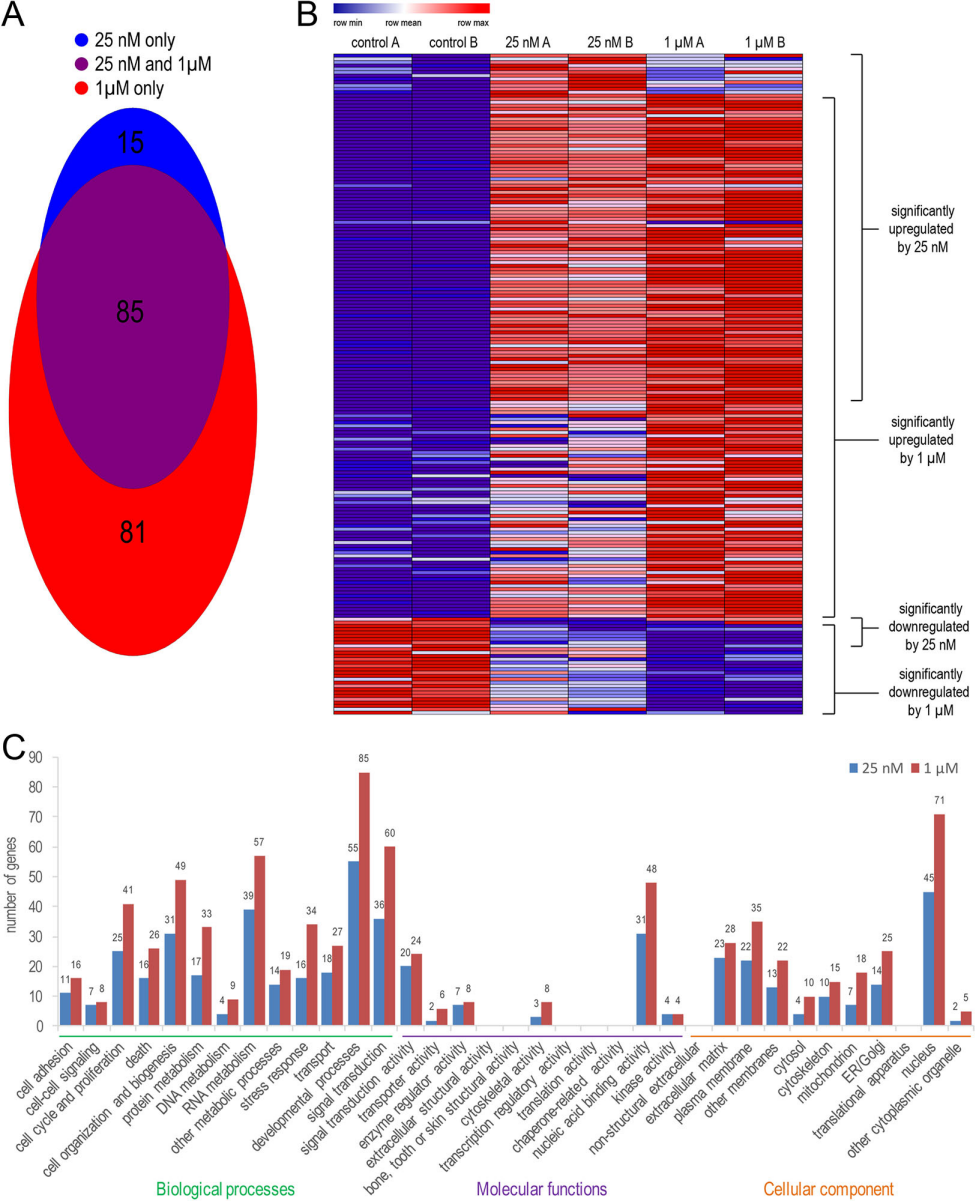
**Bioinformatic analyses of the most significantly altered transcripts in RA-treated NMPs.** The results here are based on comparisons between NMPs treated with 25 nM RA or DMSO control for 2 h, and 1 µM RA or DMSO control for 2 h, with two replicates (*n*=2) for each condition subjected to RNA-seq analysis. (A) Venn diagram depicting the amount of overlap among the transcripts altered by 25 nM and 1 µM RA. (B) Heat map analysis showing the duplicate RNA-seq results of the combined 181 transcripts significantly altered in NMPs treated with 25 nM RA (25 nM A and B) and 1 µM RA (1 µM A and B) compared to DMSO (controls A and B); brackets on right indicate genes that are upregulated or downregulated by either 25 nM RA or 1 µM RA. (C) GO analysis showing the number of transcripts altered by either 25 nM or 1 µM RA that fall into various categories of biological processes, molecular functions, and cellular components.

For our RNA-seq results, we ranked transcripts using three criteria: transcript abundance (RPKM), statistical significance (false discovery rate; FDR), and fold-change (logFC). We compiled a list of the most significantly altered transcripts; for genes activated by RA the criteria were set as RPKM>1, FDR<0.1, logFC>1, whereas for genes repressed by RA the criteria were set as RPKM>7, FDR<0.1, logFC>−0.6. No repressed gene hits were yielded under the 25 nM RA conditions with the thresholds set for gene activation, likely due to the short time period involved. We therefore lowered our threshold for logFC, while also tightening the threshold for RPKM values as compensation, using the expression parameters of *Fgf8* as a guide as it is known to be directly repressed by RA in the caudal region of mouse embryos (Kumar and Duester, 2014). Genes are listed based on FDR values with the most significant hits on top (Table S1). Under RA treatment conditions, many more transcripts were differentially expressed with 1 µM RA (166 transcripts) compared to 25 nM RA (100 transcripts), although a significant number of transcripts (85) were altered similarly by both conditions (Fig. 2A). Heat map analysis of the combined 181 significant hits obtained with 25 nM RA and 1 µM RA demonstrated that treatments with 25 nM RA and 1 µM RA result in noticeably different patterns of altered transcript abundance compared to the DMSO control or compared to each other (Fig. 2B). GO analyses of the datasets show that 25 nM RA and 1 µM RA treatments both effect the same wide array of biological processes, molecular functions, and cellular components, but with 1 µM RA consistently altering more transcripts in each category (Fig. 2C).

**Fig. 3.**
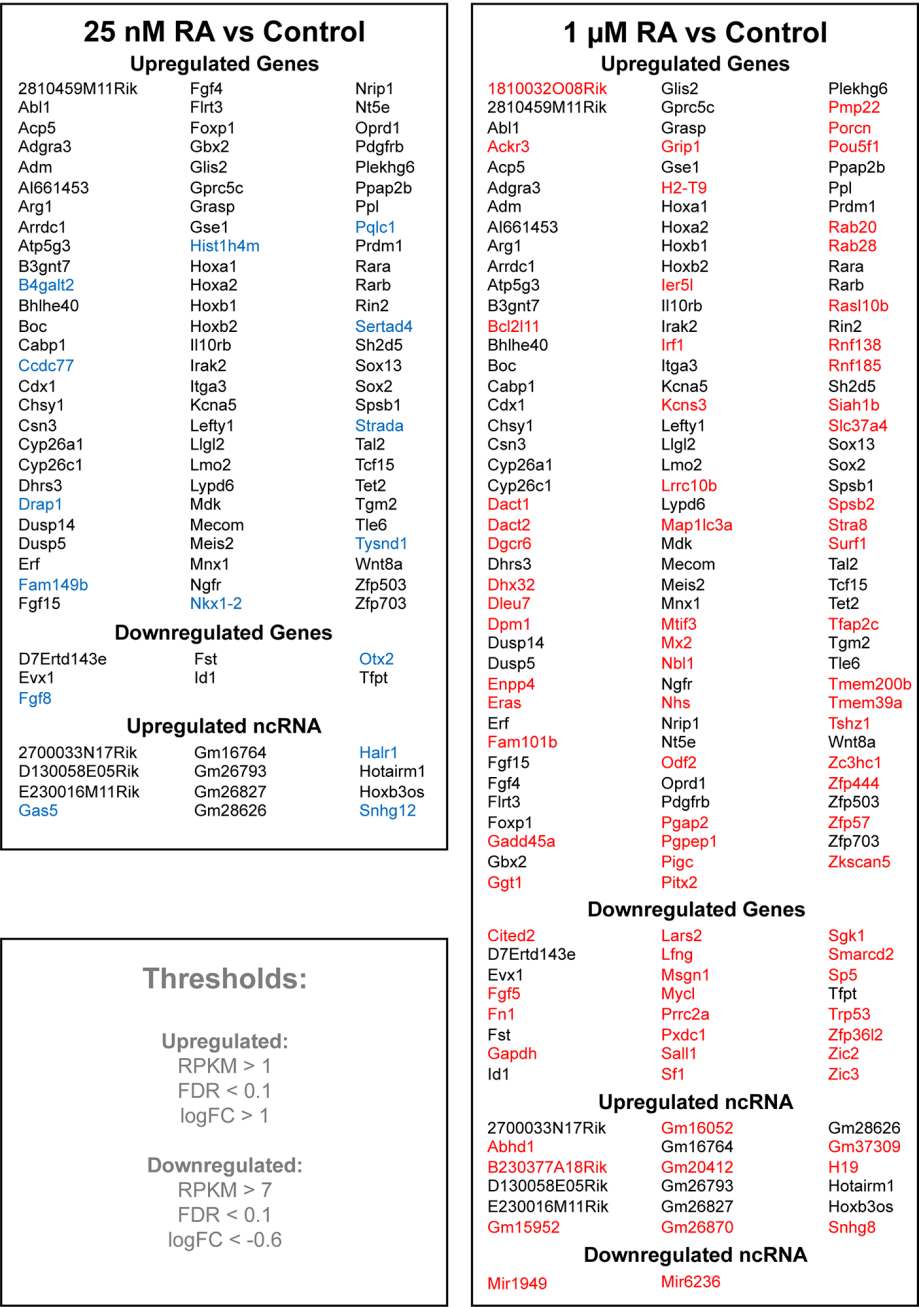
**Comparison of NMP transcripts altered by treatment with low versus high RA levels.** A list of the most significantly altered transcripts from RNA-seq analysis of RA-treated NMPs based on statistical thresholds for upregulation and downregulation shown in the lower left panel (*n*=2). Transcripts shown in blue represent those with significant changes in expression at 25 nM RA but not 1 µM RA, whereas those shown in red represent those with significant changes in expression at 1 µM RA but not 25 nM RA. Transcripts shown in black have significant changes in expression under both conditions.

We complied an alphabetical list of the transcripts significantly altered by treatment of NMPs for 2 h with either 25 nM RA or 1 µM RA (Fig. 3). Included in the list of genes differentially expressed at 1 µM RA but not 25 nM are genes that are known from *in vivo* studies to not be expressed in caudal progenitors, but that are instead known to be activated by RA in other tissues at later stages of development, i.e. *Stra8* activated by RA in spermatocytes postnatally (Raverdeau et al., 2012) and *Pitx2* activated by RA in perioptic mesenchyme at E12.5 (Kumar and Duester, 2010). The presence of RAREs near *Stra8* and *Pitx2* suggests that treatment of NMPs with 1 µM RA overrides other cues normally needed for tissue-specific expression, whereas the endogenous RA condition we employed (25 nM RA) provided a more physiologically relevant result as these genes were not significantly activated.

Conversely, we also observed genes activated at 25 nM that were not significantly activated at 1 µM RA (Fig. 3). This list includes genes that may be expected to play a role in the NMP niche, including *Nkx1-2* which is a marker of caudal progenitors and functions to activate expression of *Fgf8* and *T* (Delfino-Machin et al., 2005; Tamashiro et al., 2012; Sasai et al., 2014), as well as *Drap1* which represses Nodal signaling and is required for normal primitive streak formation (Iratni et al., 2002). We also observed genes repressed at 25 nM that were not significantly repressed at 1 µM RA (Fig. 3), including *Fgf8* which is known to be directly repressed by RA in neural plate (Diez del Corral et al., 2003; Kumar and Duester, 2014; Cunningham et al., 2015a), and *Otx2* which is downregulated in the caudal neuroectoderm (Li and Joyner, 2001). These observations provide further evidence that use of a supraphysiological dose of RA prevents a normal RA response possibly due to off-target effects.

Under both RA treatment conditions, we yielded a cohort of RA-activated, non-coding RNA genes (Fig. 3). Analysis of these reveal that most are linked to coding genes that also appear in our candidates list; those activated at 25 nM are: *D130058E05Rik* (*Gbx2*), *Hotairm1* (*Hoxa1*), *Gm16764* (*Nkx1-2*), *Gm26827* (*Chsy1*), *Gm26793* (*Fgf15*), *Gm28626* (*B3gnt7*), *2700033N17Rik* (*Meis2*), and *Halr1* (*Hoxa* cluster).

### Candidate genes that respond to treatment of NMPs with 25 nM

Going forward with our 25 nM RA condition as a more physiologically relevant result for our RNA-seq studies, we noticed that several of the top-ranked hits for highest fold-change are currently recognized RA target genes that act in the caudal region during axial development, including RA signaling and metabolism pathway genes, 3′ *Hox* genes, *Hox*-associated long non-coding RNAs, *Cdx1*, and *Fgf8*, thus validating our approach. Among the 100 top-ranked RA-responding genes, 16 have been previously examined in either RA-deficient *Raldh2*^−/−^ embryos at E7.5-E8.5 (the time period when NMPs first arise and begin to differentiate), or in RA-deficient chick or zebrafish embryos at the equivalent stage. Those published results show that 12 out of these 16 genes are validated RA target genes due to altered expression *in vivo* in the NMP niche, thus providing further evidence that our 25 nM RA dataset represents a useful list of RA target genes relevant to NMP biology (Table 1). However, the presence of four genes in this list that do not show altered expression in embryos demonstrates that *in vivo* validation is necessary to identify authentic RA target genes.

**Table 1.**
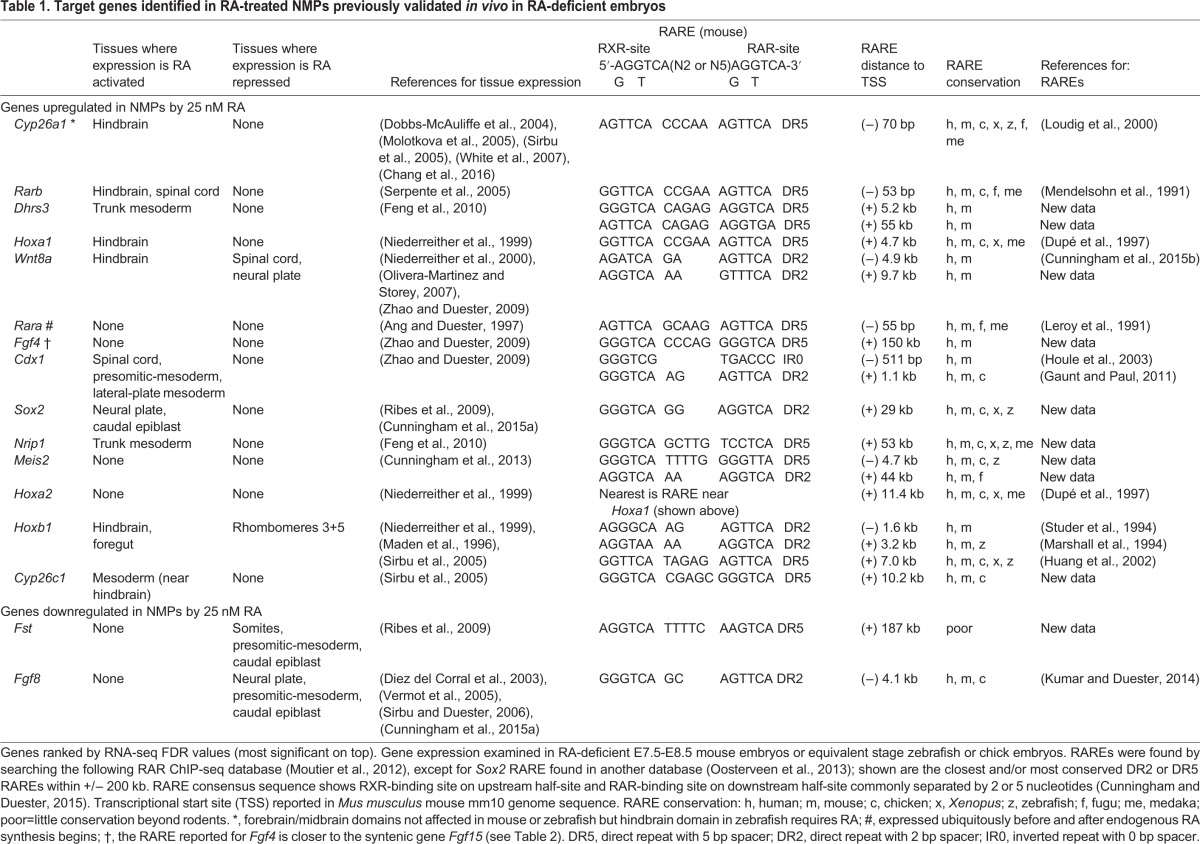
**Target genes identified in RA-treated NMPs previously validated *in vivo* in RA-deficient embryos**

Among these 16 genes previously examined in RA-deficient embryos, nine have been reported to have nearby RAREs in the mouse genome (Table 1). We further examined these RAREs and now report their locations relative to the transcription start site of the RA-responsive gene, plus we report that each of these nine RAREs is conserved in human and often in chick, *Xenopus*, or zebrafish (Table 1). Through analysis of previously published RAR ChIP-seq database for mouse embryoid bodies (Moutier et al., 2012), we identified mouse RAREs near the other seven genes previously examined in RA-deficient embryos. All these RARES are conserved in at least human, except for *Fst* which was found only in mouse (Table 1). Also, all were of the DR2 or DR5 variety (direct repeats with spacers of 2 or 5 bp) which are the most common RAREs associated with genes that are known to require RA for correct expression *in vivo* (Cunningham and Duester, 2015). These RARE analyses provide further support for the identification of RA target genes.

We next sought to examine several additional potential RA targets among this list of 100 candidates to further test our framework. From our 25 nM RA dataset, we selected nine sample RA-activated candidates across a range of average expression values (Table 2): *Zfp503*, *Zfp703*, and *Adgra3* (low RPKM 2-14.99); *Gbx2* (medium RPKM 15-39.99); *Fgf15*, *Erf*, *B3gnt7*, and *Nt5e* (high RPKM 40-74.99); and *Nkx1.2* (very high RPKM>75). We also selected two RA-repressed candidates: *Fst* (with the highest RPKM value before RA treatment, 29.2) and *Id1* (average RPKM before RA treatment, 16.1). *Zfp503*, a zinc finger gene (Ji et al., 2009) and *Gbx2*, a homeobox gene (Li and Joyner, 2001), are mentioned in the literature as being upregulated in posterior neuroectoderm using RA agonist or antagonist treatments at late mouse embryo stages (>E9.5). *Erf*, encoding an ETS repressive factor, was previously shown to be activated by RA treatment in *Xenopus* embryos (Janesick et al., 2013). Also, one gene that was previously shown to be downregulated by RA, i.e. *Fst* encoding the BMP antagonist follistatin, was re-examined here (Ribes et al., 2009). Of the remaining genes, *Nkx1-2* is expressed at a high level in the caudal region of late gastrulation embryos and at a lower level in the posterior neural tube, and is considered a key marker of NMPs (Delfino-Machin et al., 2005; Tamashiro et al., 2012; Sasai et al., 2014), *Zfp703* is a close homologue of *Zfp503*, *Fgf15* encodes fibroblast growth factor 15, with a function in neural progenitor cell differentiation (Parchem et al., 2015), *Adgra2* (Gpr124) encodes an adhesion G-protein coupled receptor needed for central nervous system angiogenesis (Kuhnert et al., 2010), *B3gnt7* encodes β-1,3-N-acetylglucosaminyltransferase, with no previous link to NMP or neural development, *Nt5e* encodes ecto-5′-nucleotidase and was previously found to be upregulated in ESC-derived NMPs (Gouti et al., 2014), and *Id1* encodes a helix-loop-helix transcription factor involved in lateral plate mesoderm development that is induced by bone morphogenetic protein (BMP) signaling in ESCs (Hollnagel et al., 1999). qRT-PCR analysis of NMPs treated with 25 nM RA for 2 h was performed to validate these 11 top hit genes as RA target genes. All were validated as RA target genes with the exception of *Erf* whose expression followed the same trend as seen in its RNA-seq results but was not significant (Fig. 1C).

**Table 2.**
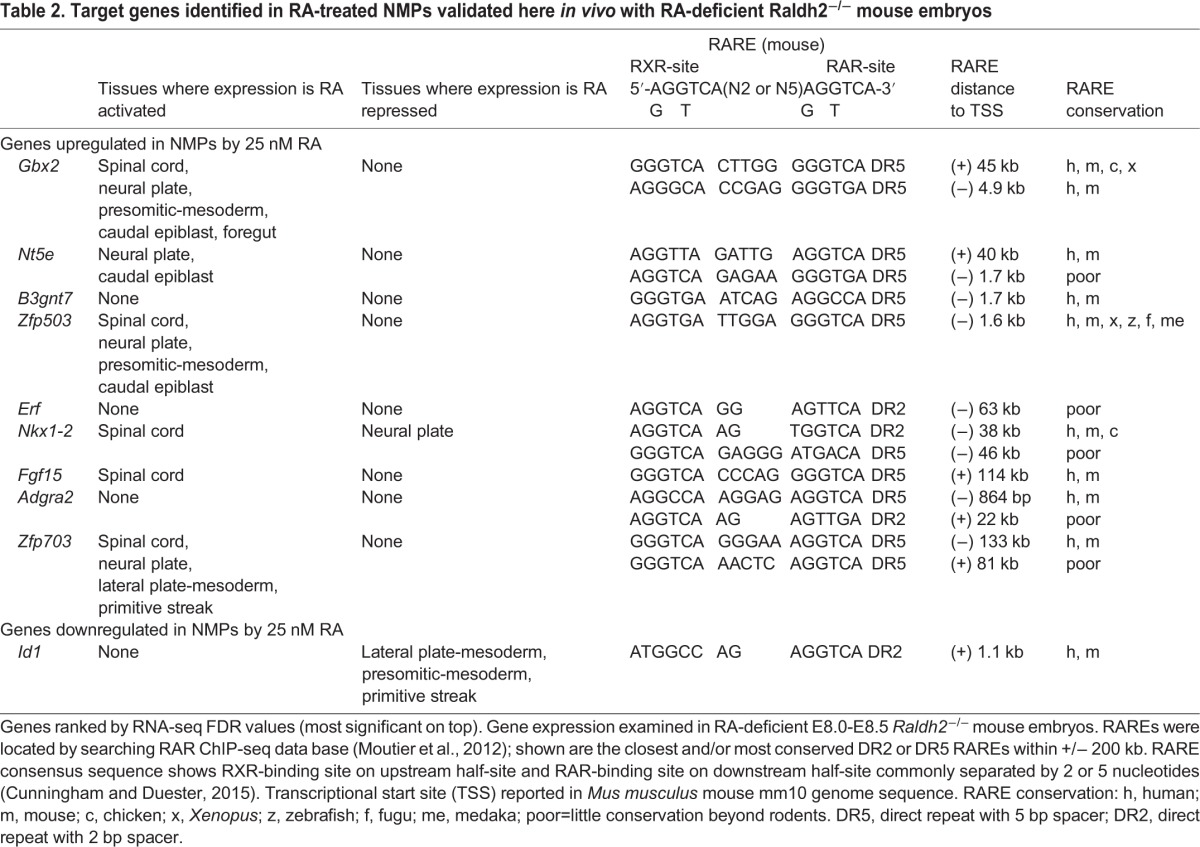
**Target genes identified in RA-treated NMPs validated here *in vivo* with RA-deficient Raldh2^−/−^ mouse embryos**

None of these 11 genes have been reported to have RAREs nearby. Through analysis of previously published mouse RAR ChIP-seq data (Moutier et al., 2012), we identified DR2 or DR5 RAREs near all 11 of these new candidate RA target genes in the mouse genome, with all being conserved at least in human with the exception of *Erf* (Table 2). Together, our RNA-seq data on RA-treated NMPs and our identification of RAREs near each of these 11 genes suggests that they are good candidates to further examine *in vivo* as potential RA target genes.

### *In vivo* validation of RA target genes in the NMP niche using *Raldh2*^−/−^ embryos

**Fig. 4.**
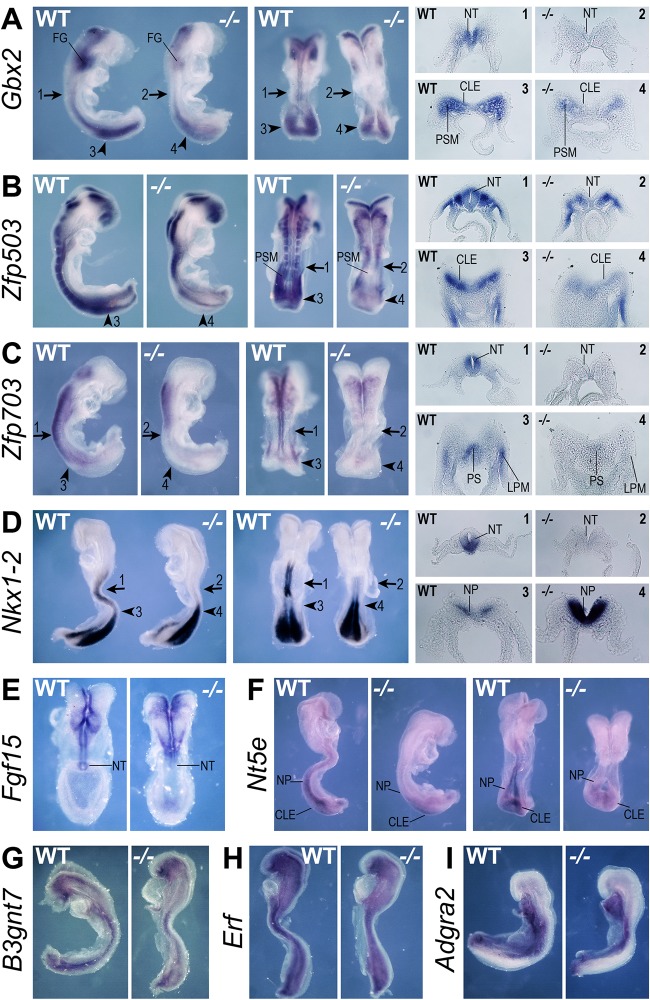
***In vivo* validation of genes activated in RA-treated NMPs.** Shown are whole-mount *in situ* hybridization results to detect mRNA in E8.0-E8.5 wild-type (WT) and *Raldh2*^−/−^ (−/−) RA-deficient mouse embryos. Analyzed here are several genes that were found by RNA-seq to be activated by treatment of NMPs with 25 nM RA: (A) *Gbx2*, (B) *Zfp503*, (C) *Zfp703*, (D) *Nkx1-2*, (E) *Fgf15*, (F) *Nt5e*, (G) *B3gnt7*, (H) *Erf*, (I) *Adgra2*; *n*=3 embryos examined for each gene. Arrows indicate the location of transverse sections through the posterior neural tube; arrowheads indicate the location of transverse sections through the caudal epiblast or neural plate; numbered panels to right refer to corresponding sections. CLE, caudal lateral epiblast; FG, foregut; LPM, lateral plate mesoderm; NT, neural tube; NP, neural plate; PS, primitive streak; PSM, presomitic mesoderm.

In order to determine whether the potential new RA-target genes identified above by RNA-seq and confirmed by qRT-PCR are regulated by RA in the NMP niche *in vivo*, we assessed their expression patterns during late gastrulation in mouse embryos by whole mount *in situ* hybridization. By comparing expression in wild-type embryos and *Raldh2*^−/−^ embryos that are deficient in synthesis of endogenous RA, we identified candidates that are affected by loss of RA signaling *in vivo*. Among the nine newly examined genes that are activated by RA in NMPs *in vitro*, we found that *Gbx2*, *Zfp503*, *Zfp703*, *Nkx1-2*, *Fgf15* and *Nt5e* exhibited decreased expression in the NMP niche (caudal epiblast, primitive streak) or immediate NMP progeny, whereas expression of *B3gnt7*, *Erf* and *Adgra2* did not change in *Raldh2*^−/−^ embryos; *n*=3 for each gene examined (Fig. 4; Table 2). *Gbx2* had greatly reduced expression in the presomitic mesoderm, spinal cord and neural plate; in caudal lateral epiblast, wild-type expression of *Gbx2* along the border with the presomitic mesoderm was lost (Fig. 4A). *Zfp503* had greatly reduced expression in the caudal lateral epiblast and presomitic mesoderm, and missing expression in neural plate/spinal cord (Fig. 3B). *Zfp703* had missing expression in the primitive streak region, neural plate/spinal cord and posterior lateral plate mesoderm (Fig. 4C). *Nkx1-2* had missing expression in the spinal cord (posterior neural tube), but expression was increased in the neural plate (Fig. 4D). *Fgf15* had missing expression in the posterior spinal cord close to the node (Fig. 4E). *Nt5e* had reduced expression in the neural plate and caudal lateral epiblast (Fig. 4F). Thus, we have identified six rapidly responding RA target genes that require RA *in vivo* for normal activation in the NMP niche and/or NMP progeny.

**Fig. 5.**
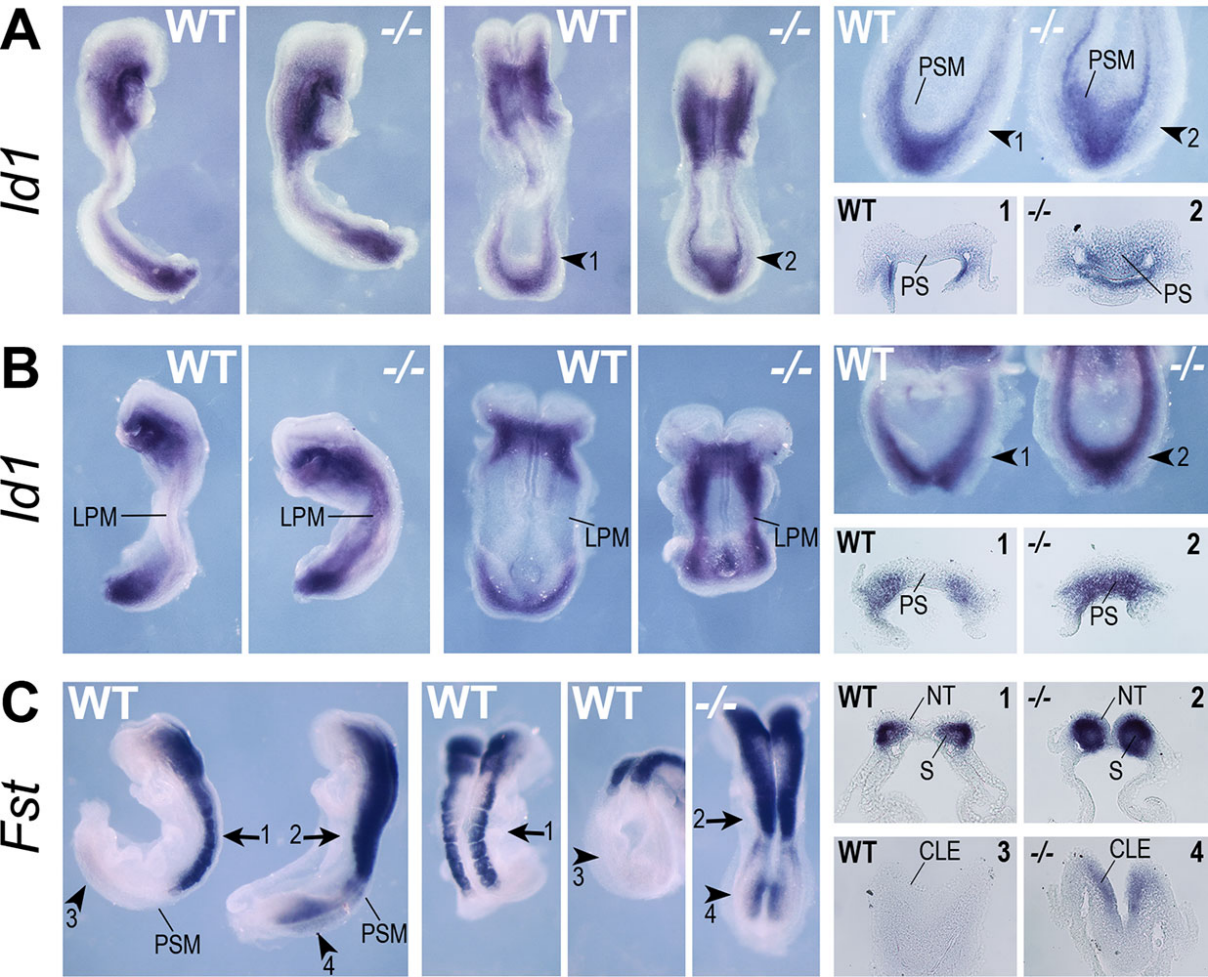
***In vivo* validation of genes repressed in RA-treated NMPs.** Shown are whole-mount *in situ* hybridization results to detect mRNA in E8.0-E8.5 wild-type (WT) and *Raldh2*^−/−^ (−/−) RA-deficient mouse embryos. Analyzed here are two genes that were found by RNA-seq to be repressed by treatment of NMPs with 25 nM RA: (A,B) *Id1*, (C) *Fst*; *n*=3 embryos examined for each gene. Arrows indicate the location of transverse sections through the posterior neural tube; arrowheads indicate the location of transverse sections through the caudal epiblast; numbered panels to right refer to corresponding sections. CLE, caudal lateral epiblast; LPM, lateral plate mesoderm; NT, neural tube; PS, primitive streak; PSM, presomitic mesoderm; S, somite.

Analysis of two potential RA target genes that are repressed by RA in NMPs (*Id1* and *Fst*) showed that both exhibit increased expression in the NMP niche when comparing wild-type and *Raldh2*^−/−^ embryos; *n*=3 for each gene examined (Fig. 5A-C; Table 2). Caudal expression of *Id1* is normally limited to the lateral/ventral mesoderm progenitors in the very caudal tip of the embryo that generate lateral plate mesoderm, however in the absence of RA we observed that *Id1* expression is extended anteriorly, ectopically encroaching into the primitive streak and presomitic mesoderm domains (Fig. 5A,B). Also, as embryos undergo body axis extension, *Id1* is normally downregulated in the trunk lateral plate mesoderm, but this process was perturbed in the absence of RA, most notably in early somite stages (Fig. 5B). Caudal expression of *Fst* is normally most evident in the somites at the 7-somite stage, however in the absence of RA we found that *Fst* is ectopically expressed in the caudal lateral epiblast and presomitic mesoderm, plus at a higher level in somites (especially in the ventrally located sclerotome) (Fig. 5C). *Fst* is normally evident in the caudal lateral epiblast at E8.0, where RA was previously shown to repress it in anterior/lateral domains of the niche (Ribes et al., 2009). We show here that RA is ultimately required to extinguish *Fst* in the whole caudal region shortly after somitogenesis begins. Thus, we have identified two RA targets that are repressed by RA in the NMP niche, in addition to two previously identified RA-repressed genes (*Fgf8* and *Wnt8a*). Together with our identification of six new RA-activated genes in the NMP niche, these observations demonstrate the effectiveness of combining *in vitro* and *in vivo* studies to identify new RA target genes.

## DISCUSSION

Previous *in vivo* genetic experiments have revealed roles for RA signaling in both posterior neurogenesis and somitogenesis, however, the role of RA within the NMP niche and the early molecular response to RA signaling is unclear. To address this, we devised an unbiased platform to study RA signaling at the whole genome level in ESC-derived NMPs with *in vivo* genetic corroboration. NMPs were treated with either a physiological dose of RA (25 nM) or a pharmacological dose of RA (1 µM) followed by whole transcriptome RNA-seq analysis. Both doses of RA activated numerous genes associated with RA signaling and metabolism, as well as other known embryonic RA signaling targets (e.g. 3′ *Hox* genes, *Cdx1*). Even within a short treatment time period, 1 µM RA altered expression of 66% more genes than 25 nM RA, including *Stra8* and *Pitx2* that are normally RA-activated but only at later stages in other tissues not derived from NMPs. Thus, 1 µM RA overrides the normal control mechanisms for many genes. Also, only about half of the genes altered by 1 µM RA were similarly altered by 25 nM RA. Our observations lead us to conclude that use of a supraphysiological dose of RA leads to inappropriate gene activation/repression, thus pointing out the importance of using endogenous RA concentrations when treating cells *in vitro* to more closely mimic *in vivo* conditions. We also note that our use of RA-treated NMPs that are exposed to RA *in vivo*, rather than ESCs that are not exposed to RA *in vivo*, is more likely to identify genes that are normally regulated by RA *in vivo*.

**Fig. 6.**
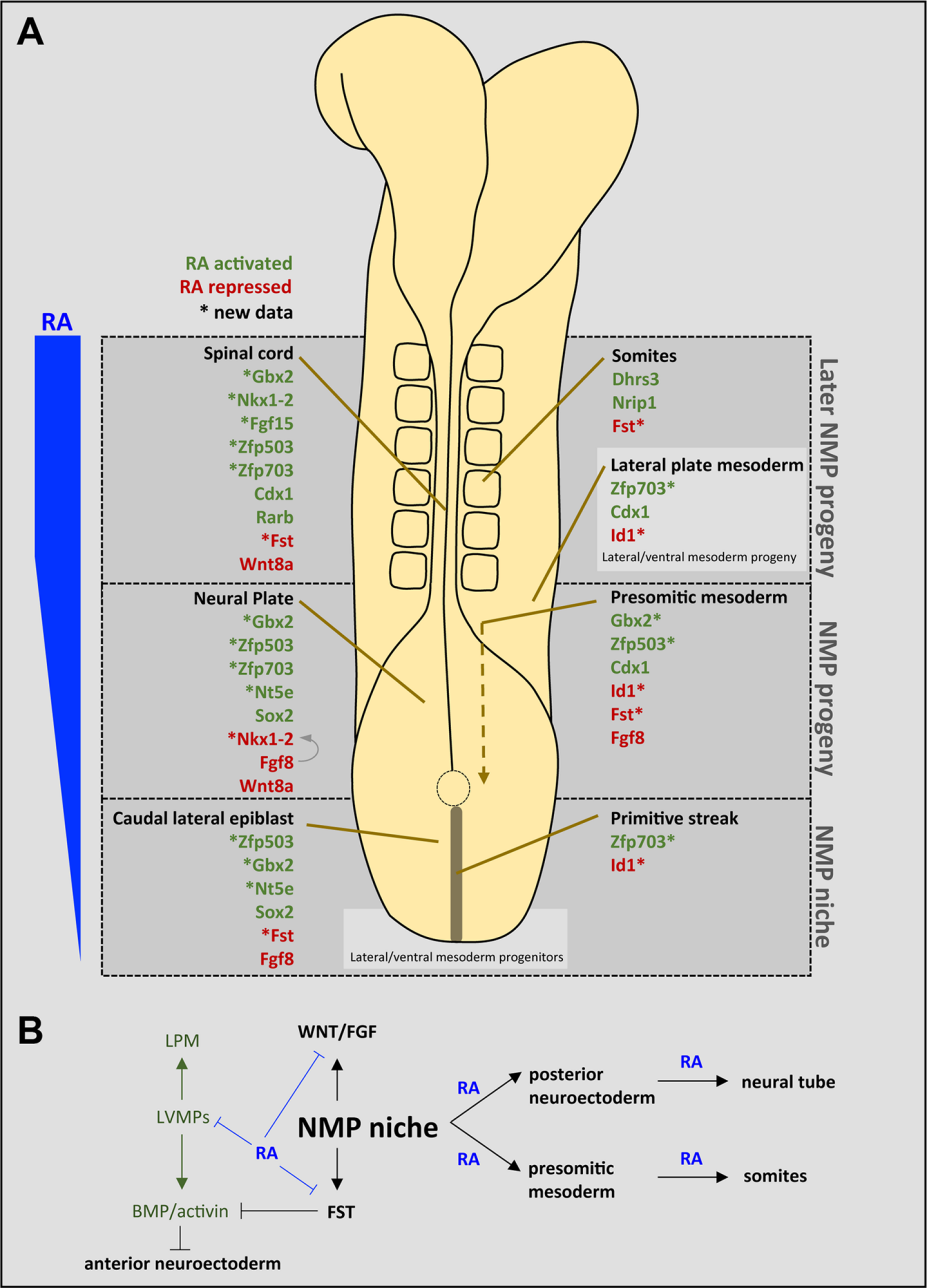
**Summary of rapidly responding RA target genes in ESC-derived NMPs validated *in vivo*.** (A) Schematic dorsal view of a 6-somite mouse embryo showing genes activated (green) or repressed (red) by RA based upon both RNA-seq data of RA-treated NMPs and RA-deficient embryos. New RA target genes identified here (marked by an asterisk) were examined in *Raldh2^−/−^* mouse embryos; remaining genes were previously shown to be RA targets based upon examination of zebrafish, chick, or mouse RA-deficient embryos. RA (indicated in blue) is produced anterior to the NMP niche, predominantly by mesodermal progeny of NMPs (presomitic mesoderm and somites that express *Raldh2*), forming a gradient that extends caudally into the NMP niche. (B) Proposed influence of RA signaling in caudal progenitors, including the NMP niche (and progeny) and lateral/ventral mesoderm progenitors (LVMPs).

Based on our 25 nM RA-treatment data, we have identified a cohort of genes expressed in the NMP niche and in NMP progeny that are rapidly activated or repressed by endogenous RA signaling, suggesting both instructive and permissive roles for RA in NMP differentiation. Combined with *in vivo* validation using RA-deficient *Raldh2*^−/−^ embryos, our findings provide new insight into NMP biology (Fig. 6). In the caudal lateral epiblast and primitive streak, together comprising the NMP niche (Wymeersch et al., 2016), expression of *Zfp503* and *Zfp703* (a pair of orthologous zinc finger proteins), *Gbx2*, and *Nt5e* was missing or greatly downregulated in the absence of RA, while *Fst* and *Id1* were upregulated. As *Raldh2*^−/−^ embryos exhibit premature termination of body axis extension (Cunningham and Duester, 2015), these observations provide candidate RA target genes for determining how RA controls maintenance of caudal progenitors. In the neuroectodermal progeny of NMPs (neural plate and spinal cord), expression of *Zfp503*, *Zfp703*, *Nt5e*, *Gbx2* and *Fgf15* was missing or greatly downregulated. In the presomitic mesodermal progeny of NMPs, expression of *Gbx2* and *Zfp503* was missing or greatly downregulated in the absence of RA, while *Fst* was ectopically expressed. Our findings thus greatly increase the number of genes known to require endogenous RA for proper expression in the presomitic mesodermal lineage (Fig. 6A). Although our observed loss of *Nkx1-2* expression in the spinal cord of *Raldh2*^−/−^ embryos is consistent with the results of our RA-treated NMPs, *Nkx1-2* expression in the neural plate of *Raldh2*^−/−^ embryos was increased by loss of RA; however, this result may be secondary to increased expression of caudal *Fgf8* in the absence of RA as FGF8 is known to activate *Nkx1-2* expression (Sasai et al., 2014). These findings suggest that *Nkx1-2* expression in caudal progenitors wanes as epiblast cells incorporate into the neural plate where less *Fgf8* is expressed, but then it is activated by RA when the neural tube is formed. Upregulation of genes expressed in the neural lineage from NMPs suggests RA is promoting early neural fate specification, consistent with impaired neural differentiation in *Raldh2*^−/−^ mutants (Cunningham and Duester, 2015). Interestingly, our 2 h RA treatment did not induce *Pax6* and *Neurog2* that possess nearby RAREs and are known to require RA for activation in the spinal cord *in vivo* (Diez del Corral et al., 2003; Molotkova et al., 2005; Ribes et al., 2008; Oosterveen et al., 2013); activation of these genes evidently occurs during later molecular events. Also, the lack of significant *Sox1* expression after 2 h of RA treatment shows that the molecular events we are observing precede mature formation of the neural tube.

Among the RA-activated neural lineage expressed genes that we find to be early responders of RA signaling in the NMP niche, several have functions later in the posterior neural lineage. *Gbx2* later functions in dorsoventral patterning of the posterior neural tube (Luu et al., 2011) and *Zfp503* has later roles during lateral motor column development (Ji et al., 2009), but a role for either in the caudal lateral epiblast or neural plate is unknown. *Zfp703* was recently identified as a negative feedback regulator of Wnt/β-catenin signaling that targets the β-catenin/TCF1 transcription complex (Kumar et al., 2016), thus potentially complementing RA repression of *Wnt8a* in the neuroectoderm (Cunningham et al., 2015b). Chick embryo studies suggest *Nkx1-2* functions to specify both floor plate and the neural crest lineages in the neural tube, whereas its function in the caudal lateral epiblast is unknown (Sasai et al., 2014). *Fgf15* functions to promote neural progenitor cell differentiation in the neural tube (Parchem et al., 2015). *Nt5e* encodes an enzyme that hydrolyzes extracellular AMP to adenosine in the spinal cord and has been identified as an NMP marker (Gouti et al., 2014), but its function in the NMP niche is unknown. From our *in vitro* analysis of RA-treated NMPs, we can now also include *Sox2* as an early upregulated responder to RA signaling during NMP differentiation; previous *in vivo* analysis of *Raldh2*^−/−^ embryos showed that *Sox2* expression in the caudal lateral epiblast and neural plate is activated by RA (Ribes et al., 2009; Cunningham et al., 2015a). Altogether, these findings demonstrate that RA promotes early neural differentiation of NMPs, i.e. the transition from caudal epiblast to neuroectoderm.

Our findings also demonstrate that RA is required for proper differentiation of NMPs to the presomitic lineage. We found that *Gbx2* and *Zfp503* (both activated by RA in the neural lineage) are also both activated by RA in presomitic mesoderm; previous studies showed that *Gbx2* functions in anteroposterior patterning of the presomitic mesoderm (Carapuco et al., 2005) whereas the function of *Zfp503* in presomitic mesoderm is unknown. These data suggest that RA activates genes to regulate the presomitic mesoderm lineage in addition to promoting neural differentiation, and in some cases the same genes are activated in both lineages (*Gbx2*, *Zfp503*). Conversely, in the absence of RA, *Id1* expression, which is normally activated by BMP in the lateral/ventral mesoderm progenitors (LVMPs) at the caudal tip of the embryo, expands anteriorly into the NMP niche and presomitic mesoderm, suggesting that RA is acting to restrict the LVMP niche, thus defining the posterior extent of the NMP niche. Recent studies have shown that LVMPs do not require WNT signaling for differentiation, whereas NMPs do, highlighting a major distinction between these two classes of caudal progenitors (Wymeersch et al., 2016). Our studies now suggest that RA functions differently in NMPs and LVMPs. Consistent with a requirement of RA to limit LVMPs, we also observed that *Id1+* lateral plate mesoderm is expanded under RA deficiency in the trunk, including enlargement of the heart. We find that *Zfp703* is activated by RA in the lateral plate mesoderm which may be associated with the ability of *Zfp703* to modulate Wnt/β-catenin signaling (Kumar et al., 2016). Thus, RA may function in both the NMP and LVMP niches to ensure balanced formation of presomitic and lateral plate mesoderm (Fig. 6B).

*Fst* is another caudal RA-repressed gene and we show that RA is required to repress *Fst* entirely in the caudal region shortly after the onset of somitogenesis, when expression shifts exclusively to the somites; thus *Fst* is an early, but not late, NMP marker and this switch is dependent on RA. *Fst* antagonizes BMP (alongside other BMP antagonists) during anterior neural induction, but posterior neural induction does not require BMP antagonism (Rentzsch et al., 2004). Thus, RA repression of *Fst* in the caudal lateral epiblast coincides with posteriorization of neural ectoderm. *Fst* also functions in the mesoderm where it promotes a presomitic mesoderm fate at the expense of a lateral plate mesoderm fate during somitogenesis (Stafford et al., 2014). We observed that RA-deficient embryos have increased *Fst* expression in both presomitic mesoderm and somites, therefore it will be interesting to determine whether increased *Fst* expression contributes to the small somite phenotype of RA-deficient embryos.

NMPs are a potentially vital source of progenitors for clinical use to treat disease or injury to the spinal cord and axial skeleton. Full understanding of the early signaling events surrounding RA-induced differentiation of NMPs is critical for understanding how neural and mesodermal tissues normally first arise and for developing successful regenerative medicine approaches based on stem/progenitor cell based therapies.

## MATERIALS AND METHODS

### Generation of NMPs and RA treatment

Mouse ESCs (C57BL/6) were maintained on mitotically inactive mouse embryonic fibroblast feeders plus ESGRO LIF (Millipore); this cell line was recently authenticated and tested for contamination. Mouse ESC differentiation to NMPs was conducted as previously described (Gouti et al., 2014; Turner et al., 2014) except for the use of retinoid-free B27 supplement that lacks both RA and retinoid substrates that could be used by the cells themselves to produce RA (retinyl esters, retinol, retinaldehyde, and carotenoids). The suppliers for critical reagents were as follows: N2 and retinoid-free B27 supplements (ThermoFisher), bFGF (PeproTech, Rocky Hill, NJ, USA), CHIR99021 (Cayman Chemical, Ann Arbor, MI, USA), all-*trans* RA (Sigma-Aldrich Corp.). Briefly, ESCs were grown at a density of 10^4^ cells cm^−2^ as adherent cultures (using gelatin-coated 6-well Corning CellBind plates) in retinoid-free N2B27 medium with bFGF (10 ng/ml) for 48 h, then with N2B27/bFGF plus CHIR (5 µM) for 24 h (or N2B27/bFGF alone to test CHIR effect). At 72 h, bFGF/CHIR99021 was removed and replaced with N2B27 plus all-*trans* RA (25 nM, 1 µM, or DMSO control), then immediately processed for total RNA extraction (mirVana kit; ThermoFisher), which was purified and stored at −80°C.

### Quantitative RT-PCR analysis of ESC-derived NMPs

For cDNA synthesis, 1 µg of purified total RNA was reverse transcribed using QuantiTect reverse transcription kit (Qiagen) according to manufacturer's instructions. qRT-PCR was conducted with the 7900HT Fast Real-Time PCR system (Applied Biosystems) using iTaq SYBR Green Supermix (Bio-Rad). Primers sequences were obtained from the PrimerBank public database (Harvard University, https://pga.mgh.harvard.edu/primerbank/) and are available upon request. Data were analyzed with the ΔΔCt method. Each condition was performed as cells grown in triplicate in three different cell culture dishes as technical replicates. The reported normalized expression values were determined by dividing the original qRT-PCR values for each replicate by the beta-actin expression values for that replicate, thus making the beta-actin value=1, then the beta-actin normalized values for each cell culture condition were divided by beta-actin normalized values for the ES cells (day 0) condition, thus making the ES cell values for each marker=1.

### RNA-seq analysis of RA-treated NMPs

Barcoded cDNA libraries were prepared from duplicate technical replicates (each condition performed as cells grown in duplicate in two different cell culture dishes) using Illumina TruSeq Stranded Total RNA w/Ribo Gold kit. Sequencing was performed on Illumina HiSeq 1500 with Rapid Run flow cell, generating 40-60 million reads per sample with 50 bp single-end reads. Sequences were aligned to mouse mm10 reference genome using TopHat splice-aware aligner (https://ccb.jhu.edu/software/tophat/index.shtml); transcript abundance was calculated using an expectation-maximization approach; reads per kilobase of genome mapped per million (RPKM) was used for within-sample normalization; generalized linear model likelihood ratio test in edgeR software (http://www.bioconductor.org/packages/release/bioc/html/edgeR.html) was used as a differential test.

Heat map analysis of RNA-seq data was performed by using the GenePattern tool from the Broad Institute (http://software.broadinstitute.org/cancer/software/genepattern). Gene ontology (GO) analysis of RNA-seq data was performed using the MGI GO Slim Chart Tool available from Mouse Genome Informatix (http://www.informatics.jax.org/gotools/MGI_GO_Slim_Chart.html).

### Generation of mouse embryos

*Raldh2*^−/−^ mice (*Mus musculus*) have been previously described (Sirbu and Duester, 2006); genotyping was performed by PCR analysis of yolk sac DNA. All mouse studies conformed to the regulatory standards adopted by the Institutional Animal Care and Use Committee at the SBP Medical Discovery Institute which approved this study under Animal Welfare Assurance Number A3053-01.

### Whole mount *in situ* hybridization

Detection of mRNA in mouse embryos by whole mount *in situ* hybridization and tissue sectioning was performed as previously described (Sirbu and Duester, 2006). RNA *in situ* probes were designed to hybridize to the last exon/3′-untranslated regions of their respective genes (with the exception of *Id1*; see below). Probes were transcribed using T7 RNA polymerase from templates generated by PCR, using T7-promoter-linked reverse primers; forward and T7-reverse primers for each gene analyzed are as follows:

*Gbx2* F AGGGCAAGGGAAAGACGAGT, T7R atgtaatacgactcactataGGCGACAGAGTACAGTGGTG; *Zfp503* F TTCATGCTCCCTAACGACCC, T7R atgtaatacgactcactataGGAGCTCAAACTAAATAATGCAC; *Zfp703* F CTGCTCAGCCATCTACGGACTC, T7R atgtaatacgactcactataGGGAAGGAAAAGGGAAGATAT; *Nkx1-2* F AGAGGAGGAGGAGGAAGCTGA, T7R atgtaatacgactcactataGGCTTGTGTGGTTGTAGTCT; *Fgf15* F GCTACTCGGAGGAAGACTGTACC, T7R atgtaatacgactcactataGGAATTCGTAATCCTAAGACC; *Nt5e* F GCGTGGTTTCTGAATACATCTC, T7R atgtaatacgactcactataGGGTACTATGGAAGGCAGAAA; *B3gnt7* F TATTGATGATGTCTTCCTGGGCA, T7R atgtaatacgactcactataGGTTTAGAGGAGTGCTGAG; *Erf* F AGGACATGAAACGGTACCTGCA, T7R atgtaatacgactcactataGGGAGAGTTGGGCTGTGG; *Adgra2* F CCGCTCCTATCCGCTCAACA, T7R atgtaatacgactcactataGGGTTTGCTGTGAACTCT; *Fst* F CAACGCTGTAATGTGGCTGTG, T7R atgtaatacgactcactataGGGACGGAAGCAGACATTTC. The *Id1* probe was generated by cloning the *Id1* cDNA coding sequence (447 bp from ATG start codon to TGA stop codon) into the pCS2+ vector, and then transcribing using T7 RNA polymerase.
